# A multilayer and spatial description of the Erasmus mobility network

**DOI:** 10.1038/s41597-020-0382-1

**Published:** 2020-02-06

**Authors:** László Gadár, Zsolt T. Kosztyán, András Telcs, János Abonyi

**Affiliations:** 1 MTA-PE Budapest Ranking Research Group, University of Pannonia, Veszprém, Hungary; 2 Institute of Advanced Studies (iASK), Koszeg, Hungary; 3 Innopod Solutions Kft., Budapest, Hungary; 4 MTA-PE “Lendület” Complex Systems Monitoring Research Group, University of Pannonia, Veszprém, Hungary; 5 Department of Quantitative Methods, University of Pannonia, Veszprém, Hungary; 6 Wigner Research Center for Physics, Budapest, Hungary; 7 VIAS - Virtual Institute of Advanced Studies, Budapest, Hungary

**Keywords:** Applied mathematics, Education, Geography

## Abstract

The Erasmus Programme is the biggest collaboration network consisting of European Higher Education Institutions (HEIs). The flows of students, teachers and staff form directed and weighted networks that connect institutions, regions and countries. Here, we present a linked and manually verified dataset of this multiplex, multipartite, multi-labelled, spatial network. We enriched the network with institutional socio-economic data from the European Tertiary Education Register (ETER) and the Global Research Identifier Database (GRID). We geocoded the headquarters of institutions and characterised the attractiveness and quality of their environments based on Points of Interest (POI) data. The linked datasets provide relevant information to grasp a more comprehensive understanding of the mobility patterns and attractiveness of the institutions.

## Abstract (summary)

**Table:** 

Measurement(s)	Socioeconomic Factors • Demographics • mobility
Technology Type(s)	digital curation
Factor Type(s)	Higher Education Institute
Sample Characteristic - Environment	city
Sample Characteristic - Location	Europe

Machine-accessible metadata file describing the reported data: 10.6084/m9.figshare.11687310

## Background & Summary

The Erasmus programme is the biggest collaboration network of European higher education institutions (HEIs). The flows of students, teachers and staff form directed and weighted networks that connect institutes, regions and countries. The properties of the institution-level collaboration network were investigated by several empirical self-assessment studies^1–3^. Regional and national connections were analyzed by gravity models^4–6^ and the generalized method of moments^7^ to explore the economic background of the connections. The structure of the network was also compared to other collaboration networks to understand the nature of such mobilities^5,8^. Only two analyses focus on the topological properties of contract- and flow- networks of the European HEIs^9,10^. We assume that this gap is due to the lack of a properly merged, standardized and cleaned database.

**Fig. 1:**
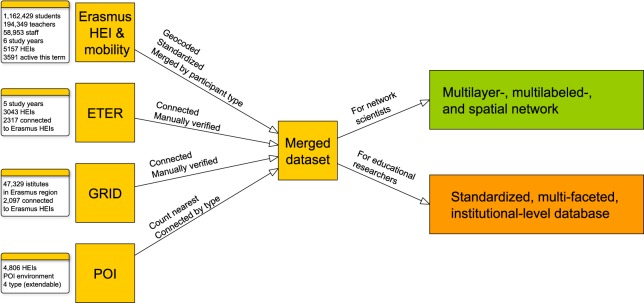
Schematic overview of the integrated higher education mobility dataset. The linked databases and their main features as well as the possibility of using the merged dataset for multiple purposes are presented on the left- and right-hand sides of the diagram, respectively.

A linked and manually verified dataset of the mobility between European higher education institutions (HEIs) as a multiplex, multipartite, multi-labelled, spatial network is presented. The dataset enriches the Erasmus mobility data with the connection between institutional variables of the European Tertiary Education Register (ETER) (https://www.eter-project.com) and the Global Research Identifier Database (GRID) (https://www.grid.ac) as well as the nearest points of interest (POI) (see Fig. 1).

This work presents the accurate identification and geocoding of the institutions, the integration of institutional- as well as regional economic data, and the manual verification of information sources. The linking of the databases greatly enriches their applicability. Erasmus data define the network of mobilities and show to what extent institutions play a sending or receiving role in the Erasmus programme. The ETER database facilitates the mobilities to be related to the number of students as well as lecturers and the budget of the institutions. The Global Research Identifier Database (GRID) links the institutions to research-related networks. The factors that determine the quality of life and tourism attractiveness of HEIs can be measured based on the points of interest (POI) dataset representing the neighbourhood of the geocoded locations.

The proposed integrated and validated network data can be used for profiling HEIs, studying socio-economic factors of mobility and measuring their attractivity based on the number of incoming students and lecturers. The understanding of the mechanisms, patterns and driving forces behind mobilities is a significant area of research as the development of integrated higher education is one of the focuses of the European Union^11,12^ and most of the higher education ranking systems take into account the internationalization of HEIs.

Mobility networks can be considered as a multidimensional network where dimensions on edges originate from categorical variables, e.g. subject area, study level of students, participant type, of the mobility database. The attractiveness of nodes can be expressed in terms of a number of socioeconomic indicators and calculated measures^13^ that require additional sources of information. The database provides the opportunity to ask such valuable research questions to which the repertoire of the multidimensional network analysis can yield valuable answers. Related analytical methods are developing very rapidly^14^. Advanced methods exist to statistically evaluate motifs^15^, communities^16,17^ and dyads^18^ of networks with categorical and continuous labels on nodes and edges^19^.

Moreover, as the geographic location of network nodes has been determined, the Erasmus exchange network has become a spatial network, therefore, its special structural features, e.g. deterrence function, distant-dependent clustering coefficient, can be detected^20^.

As the duration of the openly available mobility programme is six years, it can be interpreted as a temporal network. Although the time window of the network is too small to identify major changes in deeper socio-economic processes as the number of mobilities increases significantly every year, the analysis of this growing network might be an exciting topic of research.

## Methods

### Integration and reconciliation of heterogeneous data sources

**Fig. 2:**
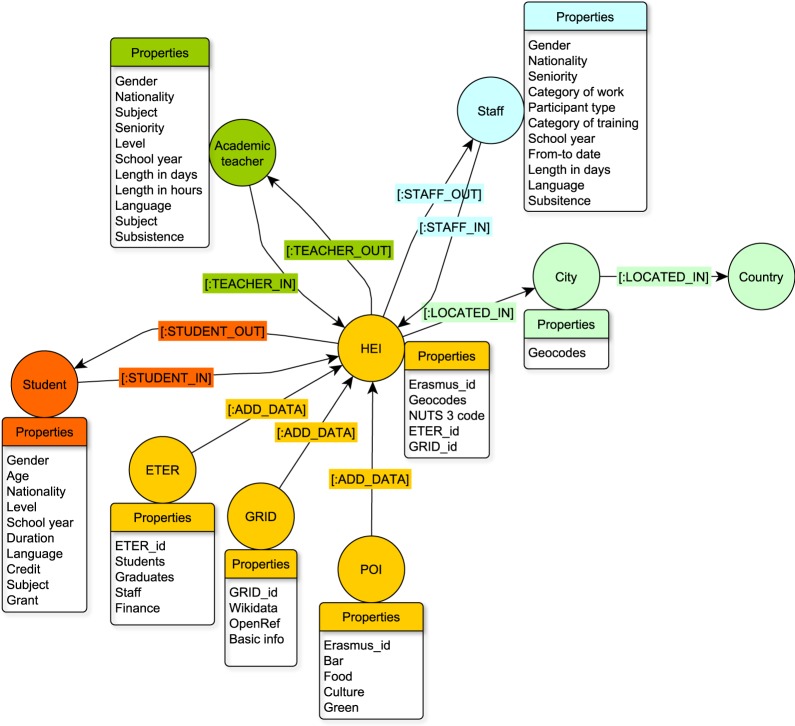
The structure of linked data.

By recognizing the need for and possibility of linking institutions in the form of multilayer spatial networks, a linked dataset, as depicted in Fig. 2, has been designed in order to determine mobility patterns at the institutional level. In the resulting network, information about edges is given from the Erasmus mobility data and the nodes are derived from various data sources. The requirement is for data sources to be as complete, comprehensive, administrative, reliable and verifiable as possible as well as openly available to researchers. The ETER, GRID and POI databases that have been chosen meet these requirements.

Linking databases and creating interfaces were major challenges. The identification of institutional data in different databases was required to achieve the connected database structure.

The Methods section is organized as follows. First, how created self-consistency for 6-year Erasmus mobility data are described. By taking into consideration the data content of the variables in different data tables, the mobility databases have been standardized and merged into one database. The unknown geographic location of Erasmus institutes brought about the need to geocode them. The next subsection discusses the geocoding procedure and counting method of different kinds of the nearest POIs that surround HEIs. Afterwards, how the interface between the institutions in the Erasmus and ETER databases was created is described. Finally, how the GRID and Erasmus institutional data is connected is described.

### Cleaning the Erasmus mobility data

Mobility databases were published annually until 2014 in the EU Open Data Portal using different data formats and headers. According to the type of participants (students, teachers, staff) three separate mobility datasets were published annually^21–26^. The procedure of cleaning and merging the data tables that cover six years of mobilities is documented in the file 1-erasmus_edgelist_merge_clean.Rmd which is an integral part of this work.

The number of rows in mobility databases was compared with official statistics from the Directorate General for Education and Culture (DG EAC) of the European Commission (see Table 1).

**Table 1:** Number of rows in mobility databases compared with official statistics.

Academic year	Type of participant	Number of records in edge databases	Number of participants according to official statistics
2008/2009	Student	167,413	168,193^31^
	Teacher	28,330	28,615^31^
	Staff	5,888	7,774^31^
2009/2010	Student	177,069	177,705^32^
	Teacher	28,772	29,031^32^
	Staff	6,910	8,745^32^
2010/2011	Student	189,914	190,498^33^
	Teacher	31,265	31,617^33^
	Staff	8,469	11,196^33^
2011/2012	Student	204,306	204,744^34^
	Teacher	32,901	33,318^34^
	Staff	9,868	13,204^34^
2012/2013	Student	211,519	212,522^35^
	Teacher	35,543	36,075^35^
	Staff	12,908	16,549^35^
2013/2014	Student	212,208	212,208^36^
	Teacher	37,538	38,108^36^
	Staff	14,910	19,380^36^
Total	Student	1,162,429	1,165,870
	Teacher	194,349	196,764
	Staff	58,953	76,848

The formation of the networked dataset continued with the consolidation of the set of HEIs. The EU Open Data Portal provides a list of HEIs that participate in the Erasmus programme but these data tables are not entirely consistent and do not contain all HEIs that send or receive any type of participant in the Erasmus mobility programme, therefore, the list of 4,919 institutions was extended by 230 HEIs that were not included in the official database but participated in the exchange programme according to the mobility databases. However, 1,566 HEIs did not send to or receive from HEIs any participants in the Erasmus programme between 2008 and 2014.

In the student mobility database 837 (0.07%) sending and 2,337 (0.02%) receiving HEIs are unknown, in the teacher mobility database 353 (0.1%) sending and 551 (0.2%) receiving HEIs are unknown, and in the staff mobility database 103 (0.1%) sending and 195 (0.2%) receiving HEIs are unknown, however, the country of origin or destination is known. To handle these connections, 31’unknown’ HEIs with the capital of the country of origin were defined and these travels remained in the mobility database. Apart from the HEis of unknown origin or destination, other information is known about travels.

Mobilities for work placement purposes have been filtered out because the destinations (companies) are very diffuse and the formulation of adequate research questions is hindered by numerous limitations.

### Geocoding and POI enrichment

The geographic location of the institutions facilitates the formation and analysis of spatial networks. The precise geocodes of HEIs enables points of interest (POI) in the neighbourhood to be searched for to characterize their social, economic and cultural environment. The number of adjacent POIs can be used as an indicator of the HEIs, to determine how they are embedded in the local economic network or how the community of HEIs generates local activities.

The manual verification of longitudinal and latitudinal information concerning HEIs in the ETER database showed that this information is inaccurate (further details in the next subsection). Moreover, not all of the Erasmus HEIs can be found in the ETER database. Therefore, even though the ETER and Erasmus HEIs databases were linked, this was unsatisfactory. As a result, all of the Erasmus institutions had to be geocoded.

The Google Places and Google Geocoding APIs services were used to harvest geocodes of HEIs. Small and closed institutes are not classified in the Google Places API, therefore, the Google Geocoding API was used with a textual search. Despite all efforts, the geocodes of 43 institutions remained unknown. These HEIs are likely to have closed or merged with others as they could not be found using Google search engine. To handle these cases, the GPS coordinates of their (and all HEIs’) city centres were also determined. Of the 43 HEIs, only 16 participated in Erasmus mobility between 2008 and 2014.

Although universities may have campuses in cities other than their headquarters, campus-relevant information is not given in the Erasmus dataset. Therefore, we fine-tuned the dataset to be coherent in terms of verifying that the places marked by the determined GPS coordinates are situated in the city of the headquarters of the universities. The assignment of exact geographic locations to institutions is essential to determine accurate inferences when using data later. Potential bias may be due to the inaccuracy of the database behind the geocoding APIs as well as changes (relocation, closure, merger) concerning institutions.

Nearby POIs were merged with Erasmus HEIs using the HERE Places (Search) API. POIs relevant to HEIs in the neighbourhood of the institutions were counted. POIs were classified as Bar (e.g. pubs), Food (e.g. fast food restaurants, bakeries), Culture (e.g. museums, theatres), Green (e.g. parks). These POIs indicate factors which complement traditional university life.

### Linking the ETER database

The European Tertiary Education Register (ETER) is a project funded by the Joint Research Centre and Directorate General for Education and Culture of the European Commission, which aims to construct a register of European HEIs and collect a comparable set of indicators and characterize HEIs according to their main activities. The partners of the project started the data acquisition process in 2013 and the project will be complemented at the end of 2019. The data is freely available for research purposes.

Annually the database provides 519 variables of the institutions, e.g. the number of students, graduates as well as academic and non-academic staff, expenditure and revenue. The quality of data and the incompleteness of the records vary from country to country. The database contains all HEIs that consist of more than 200 students and 30 full-time members of staff but excludes institutions that deliver only professional degrees (ISCED level 5).

Even though the Erasmus ID is included in the ETER database, several HEIs in the Erasmus database cannot be matched directly to the ETER dataset, as several HEIs have more than one Erasmus ID in the Erasmus database or are missing altogether. Furthermore, some of the ETER IDs had been modified over time.

By recognizing the inaccuracies in the ETER database, it was necessary to recreate the Erasmus - ETER connections. Among the methods considered, the adjacent locations were used as a primary linking procedure and the linking of textual information as a validation method.

The main uncertainty of the method is the change in the name of HEIs resulting from the merger or transformation of faculties and/or institutions. In addition,uncertainties may exist about different administrative policies on changeable higher education. Erasmus stores data on institutions as they enter the programme on a permanent basis even after a change of name. In the case of major institutional transformations, a new code is assigned to the new institution. The ETER assigns a unique identifier and annual level identifier with a year number suffix to each institutions. If a name is changed, the identifier remains unchanged, but different institutions will be associated with the annual level identifier. The GRID keeps the predecessor and successor institutions registered as a related/child/parent or alias institution along with the registration of the year of transformation. The languages of the names of HEIs also require attention as Erasmus usually uses the names in the local language. ETER has separate variables for local names and English names, and the GRID, striving for completeness, stores the local and English names as well as acronyms and related names.

ETER, as well as the Erasmus institutions, were geocoded precisely using Google APIs which provide reliable location information for historical data as well. Therefore, when developing the method to identify matched institutions, first the location information was considered followed by the names of institutions. The purely textual-based linking method, however, was able to validate the results.

The nearby locations method considers the geographically adjacent ETER institutions to the Erasmus HEIs. How similar the name of the nearest ETER institution is to each Erasmus HEI was examined using a fuzzy string matching algorithm. In the case of Erasmus HEIs that are not linked to ETER institutions, the name of the second then third nearest institutions were examined. More distant institutions were not investigated because their geographical distance is far greater than an acceptable geocoding error. At each step, manual approval and verification were performed to ensure the reliability of the resultant links.

### Linking the GRID database

The Global Research Identifier Database (GRID) is an accurate catalogue of institutions associated with academic research. As the GRID is freely accessible under a CC0 Creative Commons license, research-oriented universities can be identified and institutions connected to the world of linked open data.

As institutions that are involved in the Erasmus programme are our focus, the Erasmus HEIs and the GRID database were linked. Although HEIs in the ETER dataset were incorporated in the GRID database that was released on 2016-04-28, the links between the ETER and the GRID were not published, so linking method had to be applied to connect the two information sources.

The method used to identify the GRID institutions associated with Erasmus HEIs was similar that described in the previous subsection. The GRID records contain a wealth of metadata obtained from trusted sources. Among the metadata, the geolocation is included, so it was not necessary to geocode the GRID institutions in order to apply the nearest location method to identify matched HEIs. Institutions in the countries participating in the Erasmus programme have been included from the global data. Institutions were not filtered by type to avoid any mistakes with regard to classification in GRID database. Institutions adjacent to the Erasmus HEIs were searched for from the elements of the database thus prepared when identifying relationships.

## Data Records

The linked mobility dataset is publicly available on Mendeley Data, a secure cloud-based repository, and can be accessed directly at 10.17632/vnxdvh6998.3. The network data consist of one Excel file which defines the node set with all its attributes and linked data as well as three CSV files which describe the edge set. To supplement the data related to the nodes, POIs around the institutions and new geocodes for ETER HEIs have been published in two file^27^.

The descriptions of the nodes are found in the file 3-erasmus_HEIs.xlsx file. Microsoft Excel is capable of handling the multilingual European names of the institutions. The worksheet erasmus_hei contains the basic data of the nodes as provided in Table 2.

**Table 2:** Variables of consolidated data from Erasmus HEIs.

Variable	Description
erasmus_id	The standardized form of the Erasmus HEIs. The first three characters indicate the country. Important: countries denoted by one character contain a double space in this field.
name_local	The local name of the university.
country	The country in which the institution is located.
city	The city in which the institution is located.
lat_city	The latitudinal coordinates of the centre of the city where the headquarters of the institution is located.
lon_city	The longitudinal coordinates of the centre of the city where the headquarters of the institution is located.
lat_POI	The latitudinal coordinates of the headquarters of the institution.
lon_POI	The longitudinal coordinates of the headquarters of the institution.
name_POI	The name of the institution in the POI database.
address_POI	The address of the institution in the POI database.
NUTS3	The NUTS 3 statistical region of the European Union where the headquarters of the HEI is located.
Erasmus_original	The boolean variable that indicates whether the institute is contained in the original database provided by the EU Open Data Portal^37^. Consolidation of the node set revealed the need to supplement the database of the institution with all HEIs that are contained in the edge set.
ex_stud	The boolean variable that indicates the activity of the institution in terms of student mobility.
ex_teach	The boolean variable that indicates the activity of the institution in terms of teacher mobility.
ex_staff	The boolean variable that indicates the activity of the institution in terms of staff mobility.
ex_any	The boolean variable that indicates the activity of the institution during the 2008–2014 mobility programme between the HEIs. Participation in a trainee programme is not considered in this study.

The connections worksheet in the file 3-erasmus_HEIs.xlsx contains interfaces between other databases as described in Table 3. Some supplementary variables, which help to provide a quick overview of the value added, are included in this paper.

**Table 3:** Variables of interface data.

Variable	Description
erasmus_id	The standardized form of the Erasmus HEIs.
GRID_id	The GRID ID related to each Erasmus HEI. If this field is blank, no connected research institution exists in the GRID database.
countif_GRID	The number of Erasmus HEIs connected to the GRID institutions. More than one means that the GRID institution is connected to more than one Erasmus HEI probably as a result of a merger.
ETER_official	This variable was extracted from the ETER database which contains the Erasmus code and indicates the improvement of original open data (new connections, corrections).
ETER_geo	To ensure the transparency of this work, connections based on adjacent locations are published.
ETER_SPSS	To ensure the transparency of this work, connections based on textual similarities are published.
ETER_master	The ETER ID related to each Erasmus HEI. If this field is blank, no universities are connected in the ETER database. This variable validated based on the ETER_official, ETER_geo, ETER_SPSS variables.
ETER_master2	Additional ETER ID used when the ETER_master has been changed due to the transformation of the HEI
countif_ETER	The number of Erasmus HEIs connected to the ETER universities. More than one means that the ETER institution is connected to more than one Erasmus HEI probably as a result of a merger.
ETER_notes	Some difficulties were noted concerning transformations of the HEIs or mistakes in the official database.

The database of categorized nearby POIs are in the file 4-uni-pois.xlsx. The database of geocoded ETER institutions is accessible in the file.

5-eter-geocoded.xlsx. The ETER database is available at https://www.eter-project.com. This published file contains only the original and newly geocoded data associated with the ETER IDs and the distance between them.

The first mobility file, 6-student_merged.csv, is a semicolon-separated table, where each row of the table represents a unique student mobility. The data table contains the variables shown in Table 4.

**Table 4:** Variables of student mobility data.

Variable	Description
from_hei	The institution of origin of the student.
to_hei	The destination of the mobility.
age	The age of the student in years.
gender	The gender of the student.
nationality	The nationality of the student.
subject	The subject area of the mobility.
subject1	The main subject area of the mobility.
level	The level of study in the institution of origin.
yearprior	Number of completed years of HE study prior to the period abroad.
duration	The duration of study abroad in months.
credit	The number of credits obtained whilst studying abroad.
language	The language of study.
languageprep	The type of linguistic preparation.
sevsupp	The total value of the grant awarded for a disability over a study period. Currency in Euros.
grant	The overall subsistence expenses excluding additional funding for disabilities. Currency in Euros.
previous	This field indicates whether the student has received an Erasmus grant prior to the present one.
qualification	This field holds information on whether the student will graduate with a double or joint degree, or any other qualification at the Host Institution.
year	The academic year whilst studying abroad.
distance	The geodesic distance between the home and host cities in km, calculated based on the geocodes of the cities.
direction	The geodesic direction from the starting point to the endpoint in degrees, which was calculated based on the geocodes of the cities.

The second mobility file, 7-teacher_merged.csv, is a semicolon-separated table, where each row of the table represents a unique academic teacher mobility. The data table contains the variables shown in Table 5.

**Table 5:** Variables of academic teacher mobility data.

Variable	Description
from_hei	The institution of origin of the teacher.
to_hei	The destination of the mobility.
gender	The gender of the teacher.
nationality	The nationality of the teacher.
subjecthome	The subject area of the teacher at the home institution.
subjecthome1	The main subject area of the teacher at the home institution.
subjecthost	The subject area taught at the host institution.
subjecthost1	The main subject area taught at the host institution.
lengthdays	The number of days that the teacher has been abroad excluding travel days.
lengthhours	The number of hours the teacher taught abroad.
seniority	The degree of experience (seniority) of the teacher.
levelteaching	Level of teaching cycle at the Host Institution.
language	The language subjects are taught in the Host Country.
sevsupp	The total value of the grant awarded for a disability during a visit by a teacher. Currency in Euros.
subsistence	The overall subsistence expenses excluding additional funding for disabilities. Currency in Euros.
travel	The overall travel expenses excluding additional funding for disabilities. Currency in Euros.
isfirst	This field indicates whether this is the first teaching assignment mobility funded by Erasmus for the academic member of staff.
year	The academic year of teaching abroad.
distance	Geodesic distance between the home and host cities in km, calculated based on the geocodes of the cities.
direction	Geodesic direction from the starting point to the endpoint in degrees, calculated based on the geocodes of the cities.

The third mobility file, 8-staff_merged.csv, is a semicolon-separated table, where each rows of the table represents a unique staff mobility. The data table contains the variables shown in Table 6.

**Table 6:** Variables of staff member mobility data.

Variable	Description
from_hei	The institution of origin of the member of staff.
to_hei	The destination of the mobility.
gender	The gender of the member of staff.
nationality	The nationality of the member of staff.
workcat	The category of work at the home university.
seniority	The degree of experience (seniority) of the member of staff.
activity	The type of activity conducted by the member of staff at the host institution.
lengthdays	The number of days that the member of staff was abroad excluding travel days.
language	The language of study in the Host Country.
sevsupp	The total value of the grant awarded for a disability during a staff visit. Currency in Euros.
subsistence	The overall subsistence expenses excluding additional funding for disabilities. Currency in Euros.
travel	The overall travel costs excluding additional funding for disabilities. Currency in Euros.
isfirst	This field indicates whether this is the first staff training assignment mobility funded by Erasmus for the member of staff.
year	The academic year of training abroad.
distance	The geodesic distance between the home and host cities in km, calculated based on the geocodes of the city.
direction	The geodesic direction from the starting point to the endpoint in degrees, calculated based on the geocodes of the city.

The set of values for each variable can be found in the file 9-dictionary.xlsx. The set of values provided by the EU Open Data Portal has been standardized in order to merge different annual data.

## Technical Validation

### Validation of operations on Erasmus data

The Erasmus mobility is converted into a spatial network by geocoding the Erasmus HEIs, which is an important contribution of this work. The assignment of exact geographical locations to institutions is essential for the formation of correct inferences when using data later. The city which provided the database of Erasmus HEIs and the headquarters of HEIs were geocoded. Geocodes of cities were used as reference points to validate geocodes of the headquarters of HEIs. Geographical distances between city centres and HEIs were examined and outliers had to be investigated in detail. The location data of the cities were wrong rather than that of HEIs because the Erasmus database was incorrect, moreover, several settlements with the same or similar names in one country are present. The geocodes of cities and HEIs were carefully corrected and documented in detail in the file 5-data-validation.Rmd. Only 213 cases (4%) exist where the geographical distance between the city centre and HEI is greater than 10 km. Several incidences are present when the Erasmus database contains the nearest city but the university campus is situated far from the town. Even the universities in a big city are located in the city but far from the centre.

**Fig. 3:**
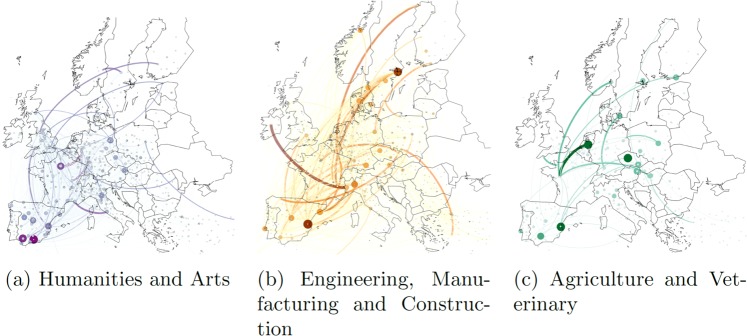
Mobility networks with different subject dimensions in the 2011–2012 academic year. The size and colour intensity of the nodes are proportional to the number of incoming students. The size and colour intensity of the edges are proportional to the number of students that travel between the connected HEIs.

As a functional validation, the network was presented on a map. Figure 3 shows the spatial multiplex structure of the student mobility network based on the determined GPS coordinates where the layers are the main subject areas of study. Some HEIs are located outside of the European continental borders, e.g. Overseas France, but these are excluded from Fig. 3.

The unknown origins and destinations remained in the tables of edges because these are valuable additional data for these trips. Table 7 shows the number of unknown origin and destination HEIs grouped by those type of the travelling and the academic year.

**Table 7:** The number of unknown HEIs in edge lists.

Year	Student		Teacher		Staff	
	origin	destination	origin	destination	origin	destination
2008/2009		16		28		2
2009/2010		80		31		8
2010/2011	421	1099	106	157	33	62
2011/2012	383	626	225	217	51	85
2012/2013	34	547	22	118	19	38
2013/2014						

Unknown institutions can be a source of some bias with regard to the structural properties of the network thus their impact have examined. The highest proportion of unknown HEIs is in the academic teacher network from 2011/2012, thus, the structure of this subnetwork was investigated. The Freeman centralizations based on the hub and authority scores are compared. For the purpose of comparability, the centralizations were normalized to the number of nodes. One of the two compared networks is the network with unknown HEIs and the other is the induced subgraph, without unknown nodes and their edges. The in- and out-degree distributions are investigated but these are not a power-law, thus, gamma exponent is not comparable. The results are shown in Table 8.

**Table 8:** Changes in centralization.

Network	Authority score centralization	Hub score centralization
With unknown nodes	0.96383	0.97211
Without unknown nodes	0.96415	0.97243

Very little difference can be observed in terms of centralization due to the small in- and out-degrees of unknown nodes. Despite the small differences between the networks with and without unknown nodes, the usage of the induced subgraph in the case of network-science based research is recommended. Due to the completeness of the data, the edges with unknown origins or destinations remained in the database.

The source of the Erasmus mobility database is based on the manual work of the national coordinators, therefore, as with any administrative database, it may contain errors. Some duplicates in the node (HEIs) set are presented, moreover, two different IDs are used for some institutions. Variables related to parameters of mobility, e.g. duration and value of the grant, may also contain errors or be ‘NA’. The correction of the wrong or imputing the missing data is impossible without knowing the data extracted from archived contracts of students, teachers and members of staff. Useful and accurate imputation techniques are exist but this work is over beyond this article. The dictionary of the mobility databases published in the EU Open Data Portal contains the sets of values for each administrative variable.

### Validation of ETER geocodes

3,043 institutions with unique identifiers are registered on the ETER database. During the validation, the geographical distances between the geocodes in the ETER database and the geocodes obtained by the API were examined. For extremely long distances (greater than 10 km), both geocodes of institutions were mapped to verify which data is correct.

In the ETER database no geocordinates are presented for 164 institutions, so new geocordinates were defined for them. The matching of the POI names returned by the API with the names of institutions was verified. When just initials of institutions were available, the validation was performed manually.

The inaccuracy of the geocodes in the ETER database is illustrated by the fact that only 172 of the 2,879 institutions posses the two types of geocode data within 50 metres and only 1,275 cases are present within 1 km. In 102 cases, the distance is greater than 100 km, which were manually verified. The institutions that are incorrectly geocoded by the API are well documented in Subsection 3.3 of the file 6-data-validation.Rmd. The resultant validated database of geocoded ETER institutions is accessible to the public as a result of this work.

### Validation of the linked ETER database

The connections between the Erasmus and ETER databases were validated by a method that is independent of the utilised location-based merging of the databases. Links between the Erasmus and ETER IDs were validated by a textual similarity-based method using SPSS Modeler Entity Analytics (SPSS-EA) and taking into account the name of the organization as well as its location (city) and country. Several open-source alternatives to SPSS-EA are available, which are implemented in R^28^) or Python^29^. However, the accuracy of these open-source alternatives is less than that of the SPSS-EA^30^.

SPSS-EA loaded both Erasmus and ETER datasets into a repository as two source tags. For all the names, locations (cities) and countries ot the organizations, features such as *frequency*, *exclusivity* and *stability* can be specified. After features have been set and datasets exported into the repository, the EA matches records of two source tags and produces an output table.

It should be noted that textual similarity-based methods are blind to institutional transformations and changes, when the name of a new HEI is changed. However, they are well suited to compare current data because the related country and city information filters out the names of the institutions that need to be compared.

Before using EA, the name of organizations was preprocessed. The official names of organizations are used in both databases. Accented letters were exchanged for ANSI characters (e.g. á ⇒ a, Ő ⇒ o) and all characters capitalized.

The *frequency* indicates how many identities can have the same value. Typically the frequency is specified to be ‘One’ for IDs, however, some times the same HEIs possessed different ETER or Erasmus IDs, therefore, in this case the frequency was set as ‘Few’ for all IDs. Since only one or a few HEIs are located in a city, the frequency of the feature of cities was set as ‘Few’, while the frequency of the feature of countries was set as ‘Many’.

The *exclusivity* indicates that an entity should typically have only one of this type of feature. For all attributes this feature was set as ‘Yes’.

The *stability* indicates the value of this feature (that is, whether it is unlikely to change during the life-time of an entity). In this case, only the country can be considered as a stable attribute. All the IDs, organization names and locations can change over time.

Although the ETER dataset contained the Erasmus IDs of the HEIs, the EA identified 68 additional matches. The SPSS-EA-based joint table also showed that in three cases the related ETER IDs were wrongly specified in the Erasmus dataset. In addition, the EA also revealed that an institution could be represented by different Erasmus IDs and ETER IDs. Following a change in ownership of HEIs, in four cases the ETER ID changed as well but the Erasmus ID remained unchanged.

The summarized results of the location-based merging method and its validation can be found in the connection worksheet of the file 3-erasmus_HEIs.xlsx. In order to ensure the transparency of this work, the Erasmus link originally included in the ETER database, moreover, the results of the location-based merging method and the textual linking method using SPSS-EA were included in the connection worksheet. In order to summarize the results, an ‘ETER_master’ variable was introduced. In the case of several Erasmus HEIs, the three types of related ETER institutions were different, thus, manual verification was required. Most of the differences were due to the transformation and merger of institutions. In the case of institutions where this procedure could not be maintained, a note is also provided in the variable ‘ETER_notes’.

Originally the ETER database included 2,251 Erasmus connections. In 25 cases, the official Erasmus link in the ETER database was found to be incorrect and 2,226 were confirmed. We uncovered an additional 262 connections using the location-based algorithm and textual similarity-based verification. We identified 194 additional links based on their locations: 3 by applying SPSS-EA, and 65 by simultaneously applying these approaches. Among the 2,483 links, we confirmed 2,235 based on their location and textual similarity. We validated the incorrect and additional connections manually by searching for the website and address of the institution concerned as well as matching its data in the ETER and Erasmus databases.

### Validation of linking GRID database

We validated the GRID-Erasmus connections by SPSS-EA based on their textual similarity. In terms of the Erasmus programme, the Erasmus name, city and country were considered during the application process, moreover, the local and English names from the ETER dataset were also taken into account. In terms of the GRID, the GRID name, city and country were considered along with aliases e.g. former names of institutions, and labels, e.g. local names of institutions in local languages. The process did not provide a conclusive link between the two databases so manual matching was also required to be comprehensive.

The result of the location-based linking method with textual similarity-based validation was an interface which expresses that an Erasmus HEI is also a research institution. The interface contains 2,219 links of which 77 are institutions that participate in the Erasmus programme but are not in the ETER database. The linking method and validation failed to yield any results for 364 HEIs that are active in the Erasmus programme and included in the ETER database presumably because, they did not exceed the threshold required to enter the GRID database.

## Data Availability

The following R and Python codes used to process the cleaning and datasets can be accessed along with their data without any restrictions. The 1-erasmus_edgelist_merge_clean.Rmd shows how datasets were cleaned and standardized. The file can be read using the freely available computer program RStudio and contains R codes of data wrangling in the form of chunks and comments about the processing steps all the way from the open datasets to the merged and cleaned data. The program 2-findplacefromtext.py helped to harvest the geocodes of the HEIs from the POI database. HEIs were also recorded as POIs in the database, so their names, addresses and coordinates could be collected. The power of the database was proven by the historical institutional data. The geographically nearest POIs, e.g. pubs and museums, to the HEIs were identified by the programme 3-POI_Here_neighbour.py. Based on the geocoordinates of the HEIs, the nearest ETER and GRID institutions that included university type institutes were matched using files 4-merge_eter_DB.Rmd and 5-merge_grid_DB.Rmd, respectively. The aim was to identify matching Erasmus and ETER as well as GRID institutions. The validation determined using R are shown in the file 6-data_validation.Rmd. All the calculations made according to the published database can be seen in the Rmd file. The Gephi software, which is popular among network scientists, can also read the data. The Network on map subsection in the file 6-data_validation.Rmd includes the data preparation steps to read three subject layers, moreover, maps in Fig. 3 are provided with the help of the file 7-network_on_map.gephi.
